# Exfoliation Syndrome and Exfoliation Glaucoma in the Navajo Nation

**DOI:** 10.3390/vision6040061

**Published:** 2022-10-03

**Authors:** Ayesha Patil, Cole Swiston, Ryan T. Wallace, Chase Paulson, Matthew E. Conley, Lori McCoy, Craig Chaya, Barbara Wirostko

**Affiliations:** 1 Department of Ophthalmology/Visual Sciences, John A Moran Eye Center, University of Utah Health, Salt Lake City, UT 84132, USA; 2 School of Medicine, University of Utah, Salt Lake City, UT 84132, USA

**Keywords:** exfoliation syndrome, exfoliation glaucoma, health disparities, epidemiology, glaucoma, pseudoexfoliation syndrome

## Abstract

(1) Background: Exfoliation syndrome (XFS) is a common cause of secondary open angle glaucoma. In 1971, Faulkner et al. estimated the prevalence of XFS among 50 Navajo Nation residents as 38%. Given that XFS can cause irreversible blindness secondary to glaucoma (XFG), this study aims to identify the current prevalence of XFS among Navajo Nation residents within the Four Corners region of the U.S. (2) Methods: A retrospective chart review was conducted from 2016 to 2021 for patients aged 18 and older. All patients with XFS or XFG diagnosed by slit lamp exam were identified through chart review. (3) Results: Of the 1152 patient charts available for review, eight patients (11 eyes) were diagnosed with XFS with three patients (4 eyes) demonstrating concomitant XFG. Within this XFS population, 50% of the patients identified as male, with a mean age of 73 years. The overall prevalence of XFS was 0.7% and the overall prevalence of XFG was found to be 0.26%. The rate of XFG among patients with XFS was 37.5%. (4) Conclusion: Compared to Faulkner’s study of Navajo Nation residents in 1971, our findings show a considerably lower prevalence of XFS at 0.7%. We present the largest study to date of XFS among this population.

## 1. Introduction

Exfoliation syndrome (XFS) is a systemic disorder characterized by the production and deposition of a characteristic fibrillar extracellular matrix in ocular tissues and other visceral organs. It was first described in 1917 by Lindberg in a Finnish population [1]. The clinical diagnosis of XFS is confirmed by the presence of grayish deposits of exfoliation material in the anterior segment of the eye, often visualized on the lens capsule or pupillary margin. Other findings and complications associated with XFS include premature cataracts [2], increased intraocular pressure and associated glaucomatous nerve damage related to exfoliation glaucoma (XFG) [3].

Since its initial description in 1917, the epidemiology of XFS has been an ensuing topic of debate in ophthalmic literature. Although it was historically thought to be most common in Scandinavian populations, it has been identified in a wide variety of populations with differences in geographic location and ethnic identity [4]. Different theories have been proposed to explain this variation including UV exposure [5,6,7], latitude of a population [7], epigenetics [8], and genetic factors [9].

The true prevalence of XFS has been difficult to identify to date, given multiple barriers in assessment [4,10]. This variability in prevalence may be due to true population differences, study sample discrepancies, variation in diagnostic criteria and inconsistencies in clinician-dependent factors, along with examination techniques, and a lack of large population-based prospective studies [3,4]. Estimates of XFS range worldwide from 0% to 38% of people over the age of 60 years. Specifically, we have seen the following population XFS prevalence reports: Greenland Inuits (0%) [11], US Louisianans (1.4%) [12], Southeastern U.S. residents (1.6%) [13], black South Africans (6–7.7%) [14], northern Chinese (5.8%) [15], Pakistani residents (6.45%) [16] and northern Sweden residents (25%) [17].

Exfoliation syndrome is the single most common identifiable source of secondary open-angle glaucoma [18]. Estimates over a decade ago on the prevalence of glaucoma in Native American populations hover around 6%; however, data are lacking regarding the burden of XFS in this population [19,20]. The most recent study by Faulkner et al. estimated the prevalence of XFS in a sample of 50 Navajo Nation residents as 38% in 1971 [21].

Outside of XFS as a risk factor for developing glaucoma, many population studies have linked this disease with various systemic co-morbidities. XFS has been associated with pelvic organ prolapse, obstructive sleep apnea, atrial fibrillation, inguinal hernias, and chronic obstructive pulmonary disease [22,23,24,25,26,27]. A greater understanding of the prevalence of XFS in this population serves to improve the evaluation and impact of systemic co-morbidities from these other related disorders. This study aims to identify the current prevalence of XFS among Navajo Nation residents within the Four Corners region of the United States.

## 2. Materials and Methods

**Figure 1:**
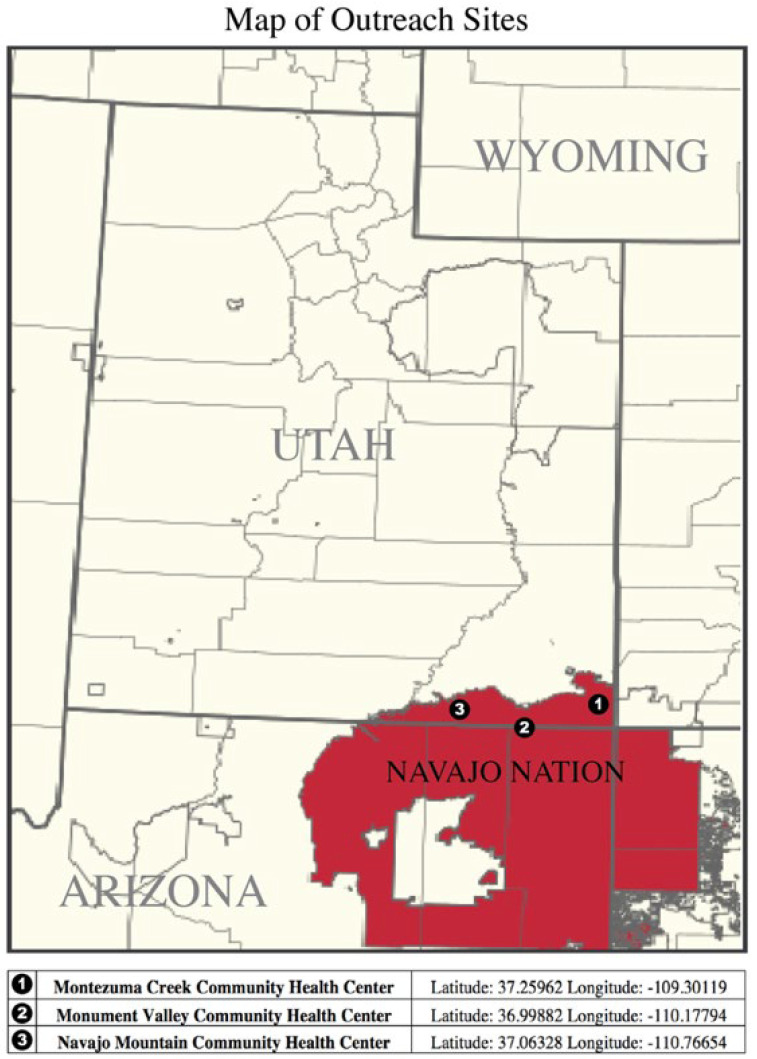
Map of outreach sites in the Navajo Nation including latitude and longitude of each site.

This study was approved by the Navajo Nation Institutional Review Board and the University of Utah Institutional Review Board (IRB_00112719). The study adhered to principles under the Declaration of Helsinki. A retrospective chart review was conducted from the period of 2016–2021 for all patients examined in partnership with the Utah Navajo Health System and the Moran Eye Center at the University of Utah. All patients are residents of the Navajo Nation and were examined at one of three sites: Monument Valley, Navajo Mountain, and Montezuma Creek (Figure 1).

Medical charts were accessed via the University of Utah Health electronic medical record (EMR). All medical information was either directly documented in the EMR during outreach clinics or later transcribed into the EMR via paper charts. All patients with XFS or XFG were identified by individual chart review. Additionally, patient demographic information and ocular/systemic comorbidities were gathered.

Exfoliation syndrome was clinically diagnosed by trained ophthalmologists using slit lamp examination. Exfoliation material was noted at the iris pupillary ruff in non-dilated patients and on the anterior lens capsule in dilated patients. Furthermore, any presence of iris transillumination defects were also noted and helped confirm the diagnosis. All XFS did not have any Krukenberg spindles noted on examination, suggestive of confounding Pigment Dispersion Syndrome. Given the patient demographics in Utah and at the Moran Eye Center, all ophthalmologists were highly skilled in clinically diagnosing XFS.

In addition to having met the criteria for XFS, patients diagnosed with XFG must have had an intraocular pressure (IOP) reading over 21.0 mmHg (measured via tonopen), optic nerve cupping (cup-to-disc ratio ≥0.5), and/or disc asymmetry, as detected during the clinical exam. Most patients were seen in-clinic for general eye screenings. All patients over the age of 18 were included in the study. All patients over the age of 50 or with a history of diabetes or glaucoma underwent a dilated eye exam, which further enabled the evaluation for XFS. Patients without a chart or visit details available on the EMR were excluded.

## 3. Results

A total of 1152 patient charts were available in our database for review from 2016 to 2021. The mean age of this population was 51.1 years with 38.2% of patients identifying as male. Of all the patients seen, 47.3% underwent a dilated eye exam (Table 1).

Eight patients (11 eyes) were diagnosed with XFS with three patients (4 eyes) demonstrating concomitant XFG. Of the patients with XFS, three had bilateral disease (XFS in both eyes), three had XFS in the right eye only, and two had XFS in the left eye only. Among XFS patients, 50.0% identified as male, with a mean age of 73.4 years. In total, all (8) of the patients with XFS underwent a dilated eye exam to confirm the presence of XFS at their recorded visit.

Of the patients with XFS, 37.5% (n = 3), 50% (n = 4), and 12.5% (n = 1) were seen at the Montezuma Creek, Monument Valley and Navajo Mountain sites, respectively (Figure 1). Average presenting IOP in XFS eyes was 19.5 mmHg (n = 11) and 18.0 mmHg (n = 4) for non-XFS fellow eyes. All patients were found to have cataracts, primarily of the nuclear sclerotic type. One patient had a unilateral nasal pterygium that shared laterality with XFS. Five patients had self-reported type II diabetes (63%), and three patients had self-reported hypertension (38%). One patient had concomitant age-related macular degeneration in the same eye as XFS (6%) (Table 2). The overall prevalence of XFS was found to be 0.7% and the overall prevalence of XFG was found to be 0.26%. Among patients 60 years or older, the prevalence of XFS was 1.8% The rate of XFG among patients with XFS was 37.5%.

## 4. Discussion

The prevalence of XFS in the literature has varied considerably among different geographic regions and ethnic populations. It has been reported as low as 0% in Inuits, and as high as 38% among Navajo Nation residents previously [11,21]. Compared to Faulkner’s study of Navajo Nation residents in 1971 among 50 patients, age 60 or older, our findings show a considerably lower prevalence of XFS at 0.7% in this larger population-based study. When directly comparing the prevalence of XFS in both studies among patients 60 years and older, our study prevalence is still significantly lower at 1.8% (7 of 389 patients). Among the patients found to have XFS in our study, none of them were related on surname identification alone. The discrepancy of our findings compared to Faulkner’s could be due to the variation in diagnostic and inclusion criteria, clinician-dependent factors, and perhaps most critically, differences in sample size and study population.

XFS is thought to be a polygenic disease, with epigenetics playing a key role. While *LOXL1* has been extensively studied, several other genes have been implicated in the pathogenesis [28]. The large discrepancy in the prevalence of XFS between our analysis and the Faulkner study could indicate unique underlying genetic factors within a single ethnic group which spans a large geographic area of the American Southwest. This has not been investigated to date.

There has been conflicting evidence in the literature to date regarding environmental risk factors for XFS. The Reykjavik Eye Study in Icelanders did not find risk associated with greater time spent outdoors [29]. However, studies in aboriginal Australians, rural residents on the island of Rab, Uyger farmers in a semiarid desert region of China, and residents with greater time spent outdoors suggest that greater ambient solar exposure is a risk factor for developing XFS [5,6,7,30,31,32]. As an agricultural and farming-based community located in the desert west, this theory would point towards the higher prevalence of XFS among the Navajo Nation population, yet our study does not support this. Given that the Navajo Nation is located closer to the equator compared to populations classically associated with XFS (e.g., Scandinavians), the geographic latitude of the Navajo reservation may be a protective factor against developing XFS according to Pasquale et al. [6].

This study was conducted by the retrospective chart review, thereby introducing limitations to the strength of our data. Notably, all charts were reviewed manually. There is also selection bias, as participants in this study opted to attend an outreach eye clinic, rather than a random sample from the community, and perhaps had more ocular complaints and or concerns. Similarly, our findings may be subject to ascertainment bias as not all patients included in the study underwent a dilated exam, and eye dilation is known to facilitate the diagnosis of XFS.

To our knowledge, this is the first study published on XFS in the Navajo Nation since Faulkner’s publication in 1971 [21]. Moreover, it is the largest study of its kind in this population with a sample size of 1152 residents who attended an outreach clinic at the three different locations. Our findings bring up many questions regarding the genetic and environmental underpinnings of XFS. Further investigation of the risk factors unique to this population may prove to therapeutically benefit both the Navajo Nation residents as well as the global population affected by this sight-threatening disease.

## 5. Conclusions

We found that the prevalence of XFS among Navajo Nation residents between 2016 and 2021 to be 0.7%. This is significantly different from Faulkner’s findings of 38% in 1971. Our findings share a similar rate to Inuit people and residents of South Louisiana, two populations who share a similar latitude [11,12].

## Figures and Tables

**Table 1:** Descriptive Summary of all Study Patients.

Variable	Result (St. Dev)
Total Patients Seen	1152
Average Age	51.1 years (17.8)
Male	440
Site of visit	
*Montezuma Creek*	487
*Monument Valley*	415
*Navajo Mountain*	250
Dilated Eye Exam Performed	545
Average IOP (mmHg)	
*Right Eye*	15.5 (4.1)
*Left Eye*	15.5 (4.6)
Diabetes Mellitus Type II	263
Hypertension	198

**Table 2:** Descriptive Summary of Patients with XFS.

Variable	Summary (N = 8 Patients) [St. Dev]
Average Age	73 years
Male	4 (50%)
Site of visit	
*Montezuma Creek*	3 (37.5%)
*Monument Valley*	4 (50.0%)
*Navajo Mountain*	1 (12.5%)
XFS Laterality	
*Bilateral*	3 (37.5%)
*Left eye*	2 (25.0%)
*Right Eye*	3 (37.5%)
Average IOP	
*Presenting Eye*	19.5 (11) [9.1]
*Fellow Eye*	18.0 (4) [4.3]
XFG	3 (21%)
Diabetes Mellitus Type II	5 (62.5%)
Hypertension	3 (37.5%)

## Data Availability

Data is available at request.
